# Compensatory Growth in Juveniles of Freshwater Redclaw Crayfish *Cherax quadricarinatus* Reared at Three Different Temperatures: Hyperphagia and Food Efficiency as Primary Mechanisms

**DOI:** 10.1371/journal.pone.0139372

**Published:** 2015-09-30

**Authors:** Liane Stumpf, Laura S. López Greco

**Affiliations:** 1 Biology of Reproduction and Growth in Crustaceans, Department of Biodiversity and Experimental Biology, FCEyN, University of Buenos Aires, Buenos Aires, Argentina; 2 IBBEA, CONICET-UBA, Buenos Aires, Argentina; University of Tours, FRANCE

## Abstract

Feeding restriction, as a trigger for compensatory growth, might be considered an alternative viable strategy for minimizing waste as well as production costs. The study assessed whether juvenile redclaw crayfish *Cherax quadricarinatus* (initial weight 0.99 ±0.03 g) was able to compensate for feeding restriction at different temperatures (23±1, 27±1 and 31±1°C). Hyperphagia, food utilization efficiency, energetic reserves, and hepatopancreas structure were analyzed. Three temperatures and two feeding regimes (DF-daily fed throughout the experiment and CF- 4 days food deprivation followed by 4 days of feeding, intermittently) were tested. The restriction period was from day 1 to 45, and the recovery period was from day 45 to 90. The previously restricted crayfish held at 23, 27, and 31 ± 1°C displayed complete body weight catch-up through compensatory growth following the restriction period with depressed growth. The mechanisms that might explain this response were higher feed intake (hyperphagia), and increased food utilization efficiency. Hepatopancreatic lipids were used as a metabolic fuel and hepatosomatic index was reduced in the previously restricted crayfish, but recovery at the same level of unrestricted crayfish occurred after the shift to daily feeding. The highest temperature affected adversely growth, feed intake, food efficiency, and metabolism of crayfish, whereas the lowest temperature and feeding restriction induced a more efficient growth of the crayfish.

## Introduction

An adequate feeding management is one of the main factors for successful farming in aquaculture, since it optimizes growth, survival and food conversion of organisms, therefore reducing the waste of food. Hence, feeding restriction, as a trigger for compensatory growth, might be considered an alternative viable strategy for minimizing waste as well as production costs.

Compensatory growth occurs in individuals when favorable conditions are introduced after a period of growth depression, and this growth is higher than that shown by individuals that have not been exposed to any unfavorable conditions [1]. Compensatory growth occurs in newly hatched and older developmental stages of redclaw crayfish *Cherax quadricarinatus*. The degree of recovery (catch-up) is dependent on the intensity of daily feeding post-restriction [2–5] and this compensatory response is achieved with increased feed utilization efficiency. Hitherto, compensatory responses have not been investigated in redclaw crayfish exposed to different temperatures despite the fact that temperature has important influences on the ability of ectothermic animals to acquire and utilize nutrients for growth [6–11].

Redclaw crayfish is a decapod crustacean endemic to freshwater habitats of northeastern Queensland, northern and eastern parts of the Northern Territory of Australia, and southeastern Papua New Guinea. It is a eurythermal species that grows well at temperatures between 24°C and 30°C, and when cultured it can reach commercial size (50–100 g) in approximately 7 months [12,13]. It is suitable for aquaculture because of its high growth rate, simple production technology, tolerance of relatively high stocking densities, and a wide range of water quality [14]. Culture of redclaw crayfish is carried out in South-East Asia and Central/South America, as well as North-Eastern Australia [15].

The aim of the present study was to assess whether redclaw crayfish are able to compensate for feeding restriction at temperatures regarded to be below and above the optimum for growth. The possibility that hyperphagia and improved food utilization efficiency were drivers of recovery growth was examined, as well as the effect of temperature on energy stores and the structure of the hepatopancreas.

## Materials and Methods

### Animals, acclimatization and culture conditions

*Cherax quadricarinatus* juveniles used in the present study were obtained under laboratory conditions, from a reproductive stock supplied by Centro Nacional de Desarrollo Acuícola (Cenadac), Corrientes, Argentina. Six ovigerous females were placed in individual glass aquaria (60×40×30 cm) containing 30L of dechlorinated water (pH 7–8, hardness 70–100 mg/L as CaCO_3_ equivalents) under continuous aeration to maintain a dissolved oxygen concentration of > 5 mg/L. Water temperature was held constant at 27±1°C and the photoperiod was 14L: 10D according to Jones (1997) [16]. These females were fed daily *ad libitum* with *Elodea* sp. and commercial balanced food for tropical fish Tetracolor, TETRA® (containing 47.5% crude protein; 6.5% crude fat; 2.0% crude fiber and 6.0% moisture). This diet is adequate for growth and reproduction of the species under laboratory conditions [17]. The incubation period varied between 30 and 45 days (27°C) and once hatched, juveniles remained with the mother, using her as a refuge until complete independence at juvenile stage III [16, 18]. After reaching the free-living stage III, juveniles were separated from each mother and maintained in glass aquaria (60×40×30 cm) containing 30L of dechlorinated water, named *growth* a*quaria* (comprising 25 juveniles/aquaria), under the same conditions of feeding, water quality, temperature, and photoperiod as described above, until reaching an average mass of 1 g (range of 0.8–1.2 g). The juveniles used in this experiment were the first brood of these females.

When the appropriate mass was achieved, a pool of these juveniles were selected and transferred individually to plastic container (9cm diameter x 7cm height, freshwater volume 500 ml) with water temperature at 27±1°C (same temperature as growth aquaria), and thereafter, juveniles used for other temperatures were acclimated to different temperatures (23 and 31 ± 1°C). In each plastic container a piece of synthetic net was provided as shelter (5cm × 5cm) with continuous aeration (Precision SR-7500 aquarium air pump). The period of acclimatization in the plastic containers was 1 week, and during this time juveniles were fed once daily. After the acclimatization period, juveniles were weighed and the experiment started. The diet used in the acclimatization and experimental periods was Tetracolor, Tetra® (as described above). Temperature was checked daily and photoperiod was 14L: 10D.

From days 1 to 45, water in plastic containers was 100% replaced every 4 days, to ensure the removal of food waste prior to food deprivation in CF treatments. Thereafter, water was replaced every 7 days.

### Experimental Procedure

**Fig 1:**
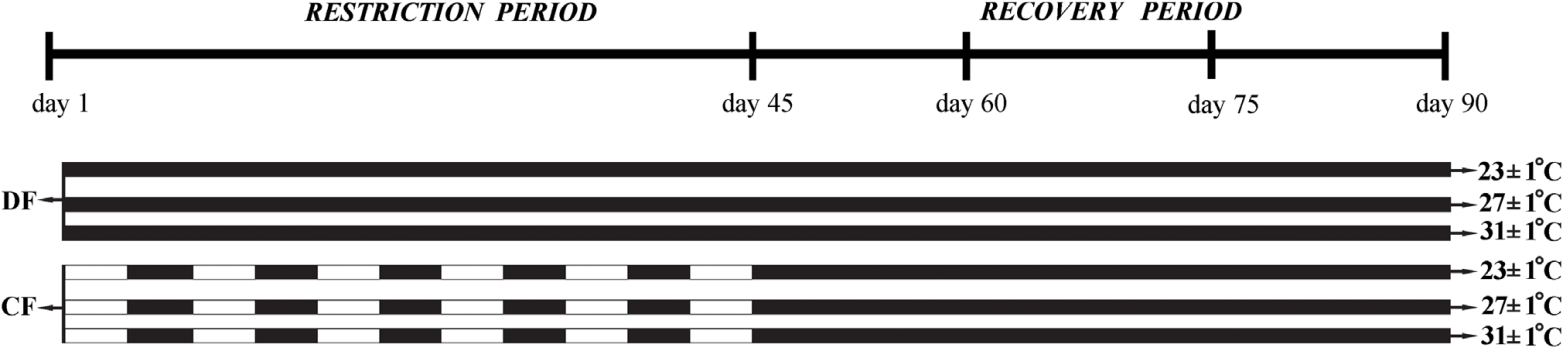
General schedule of the protocol applied in the experiment, and the treatments used in juveniles of the redclaw crayfish *Cherax quadricarinatus* Black square: Fed and White square: Unfed. Temperature regimes: 23±1°C, 27±1°C and 31±1°C and feeding regimes: DF (juveniles fed daily throughout the experimental period) and CF (juveniles fed for 4 days followed by 4 days of food deprivation, intermittently during the first 45 days of the experimental period and fed daily from day 45 to day 90).

The factorial experiment was performed by means of a 3 × 2 (three temperatures and two feeding regimes), and lasted 90 days. Three temperature regimes (23±1°C, 27±1°C and 31±1°C) that cover the optimum range for *C*. *quadricarinatus* growth [17] were chosen in this experiment; they were named low, optimum and high temperature, respectively. Two feeding regimes were established for each temperature, the control: daily fed throughout the experiment (DF), and the cyclic feeding (CF). The CF feeding regime was carried out intermittently, through 4 days of food deprivation followed by 4 days of feeding, until day 45 (restriction period). Thereafter from day 45 to day 90, juveniles were daily fed (recovery period) (Fig 1). This feeding regime was selected based on previous compensatory growth results with complete catch-up in the same species [5].

At the beginning of the experiment, three hundred juveniles weighing 0.99 ± 0.03 g (mean ± standard deviation) were assigned to one of the following treatments as shown in the general scheme of protocol and treatments used in the experiment (Fig 1). Each treatment had fifty replicates (each juvenile placed individually in a plastic container as described above). Juveniles were fed *ad libitum* during restriction period (days 1–45), and at the rate of 2% of body mass on subsequent recovery period (days 45–90). The amount of food was adjusted based on the crayfish body mass collected during each recovery period (days 45, 60 and 75).

### Sample collection and analysis

Juveniles were weighed (on wet basis) at days 1, 45, 60, 75 and 90. Each juvenile of *C*. *quadricarinatus* corresponded to a replicate for analysis of the growth, organ-somatic indexes, histological analysis and biochemical analysis of the abdominal muscle. In the case of biochemical analysis of the hepatopancreas, some juveniles (replicates) were pooled (S1 Table).

In order to analyze the organ-somatic indexes, at day one were selected 8 or 10 juveniles *per* treatment (depending on the treatment). At the end of restriction period (day 45), 9 juveniles *per* treatment (of the 50 initial replicates *per* treatment) were selected and every 15 days of recovery period (days 60, 75 and 90), 8 to 10 juveniles *per* treatment (depending on day and treatment) were selected from the animals remaining in each treatment. All these juveniles were sacrificed after being cold anaesthetized at -20°C for 15 minutes. After sacrifice, the hepatopancreas and pleon from each juvenile were rapidly dissected and weighed on an analytical balance (Pioneer^TM^,Ohaus) precision 0.1 mg for calculation of the organ-somatic indexes.

For histological analysis, a small piece of each hepatopancreas corresponding to 4 juveniles or replicates *per* treatment of those previously sacrificed for organ-somatic indexes, were fixed in Bouin’s solution. Immediately after, all tissues were frozen in a freezer at -70°C for further biochemical analysis.

For biochemical analysis of the hepatopancreas, at day 1, 45, 60, 75 and 90, a pool of juveniles or replicates was used (corresponding to juveniles of those previously sacrificed for organ-somatic indexes) in order to obtain a minimum of mass needed for these determinations.

For biochemical analysis of the abdominal muscle, at day 1, 45, 60, 75 and 90, 3 to 6 juveniles or replicates (depending of day and treatment and corresponding to juveniles of those previously sacrificed for organ-somatic indexes) was used.

The feeding intake was based on 13 or 14 animals selected (depending on treatment) at the end of the restriction period (day 45). It should be noted that as a whole there were 80 animals (adding all treatments) which had their feeding intake daily registered during the recovery period. For food efficiency calculation, feeding intake data of the selected animals (depending on treatment) were used. These juveniles accounted for more than half of the individuals which remained alive at the end of the experiment. Growth (body mass and SGR) at day 90 was based on the selected animals (depending on treatment) used for feeding intake.

For determination of total lipids, total proteins and glycogen were used methods described by Folch et al. (1957) [19] (modified by Frings et al. 1972) [20], Bradford et al. (1976) [21] and Van Handel (1965) [22], respectively. All determinations were done on wet basis and performed by triplicate (three sub-samples for each sample) using spectrophotometric methods (Jasco—UV/VIS spectrophotometer, Model 7850).

For total lipids determination, a 60 mg subsample of hepatopancreas and abdominal muscle was homogenized from a mixture of chloroform and methanol (2:1, v/v) for extraction of lipids. Subsequently, each sample was filtered, mixed and centrifuged with NaCl 0.9% to separate the lipid fraction. Afterwards, total lipids were quantified by the sulfophosphovanillin method, with olive oil (Indalo Clasico) diluted with absolute ethanol as standard. Absorbance was read at 530 nm.

For proteins determination, a 70 mg subsample of hepatopancreas and abdominal muscle was homogenized in 50mM Tris-HCl buffer, pH 7.5, and centrifuged at 10,000 *g* for 30 minutes in a cooling centrifuge (4°C). Total proteins were estimated in the supernatant by the Coomassie blue dye method, with bovine serum albumin (Fracc.V, Standard) as a standard. Absorbance was read at 595 nm.

For glycogen determination, a 50 mg subsample of hepatopancreas and abdominal muscle was boiled in KOH 30% for 20 minutes. Following separation, glycogen was precipitated with the addition of saturated Na_2_SO_4_ and ethylic alcohol at 96°, and centrifuged at 3,500 *g* for 10 minutes. Then, it was dissolved by the addition of 250 μl of distilled water, and glycogen levels were determined by the Antrhone method, with rabbit liver (Fluka) used as standard. Absorbance was read at 620 nm.

For determination of hepatopancreas histology, the samples were dehydrated in alcohol series and embedded in paraffin. Sections (6 μm thick) were stained with hematoxylin-eosin [23], and characterized based on recent descriptions of the hepatopancreas structure for the species [24].

Finally, for quantification of the feeding intake daily food supplied was weighed and recorded. Uneaten food was collected within 1h after each meal, dried in an oven at 60°C up to constant mass, weighed, and recorded again. Feeding intake was estimated through the difference in dry mass, between the amount of food supplied into the plastic container and uneaten food.

### Calculation and statistical analysis

The formulae used to calculate the different indices were as follows:

Survival:S, % = [final number of crayfish/initial number of crayfish] * 100  (1)

$$Specific\;growth\;rate:\text{ SGR},\%/\text{day }=\;\left[\left(\text{Ln}M_{90}-\text{ Ln}M_{45}\right)\;/T_{45-90}\right]*\;100\;$$ (2)

$$Hepatosomatic\;index:\text{ HSI},\;\%\;=\;\left[H_{1,45,60,75,90}/M_{1,45,60,75,90}\right]*\;100\;$$ (3)

$$Relative\;pleon\;mass:\text{ RPM},\%\;=\;\left[P_{1,45,60,75,90}/M_{1,45,60,75,90}\right]*\;100\;$$ (4)

$$Feeding\;intake:\text{ FI},\%\text{body}\;\text{mass}/\text{day }=\;\left[C/\;\left(M_{i}+\;M_{f}/2\right)*\;T\right]*\;100\;$$ (5)

$$Apparent\;food\;efficiency:\text{ AFE }=\;\left[\left(M_{90}–M_{45}\right)/C_{45-90}\right]$$ (6)

$$Protein\;efficiency\;ratio:\text{ PER }=\;\left[\left(M_{90}-M_{45}\right)/protC_{45-90}\right]\;$$ (7)

$$Lipids\;efficiency\;ratio:\text{ LER }=\;\left[\left(M_{90}-M_{45}\right)/lipC_{45-90}\right]$$ (8)

where *M*_*45*_ and *M*_*90*_ are body masses on a wet mass basis (g) at days 45 and 90, respectively, *T*_*45-90*_ is time (days) corresponding to the recovery period, *H*_*1*,*45*,*60*,*75*, *90*_ are hepatopancreas masses at days 1, 45, 60, 75 and 90, *P*_1,45,60,75, 90_ are pleon masses on a wet mass basis (g) at days 1, 45, 60, 75 and 90, *M*_*1*,*45*,*60*,*75*,*90*_ are body masses on a wet mass basis (g) at days 1, 45, 60, 75 and 90, *M*_*f*_ and *M*_*i*_ are final and initial body masses on a wet mass basis (g), *T* is time (days), *C* is food consumption on a dry mass basis (g), *C*_*45-90*_ is food consumption on a dry mass basis (g) during the recovery period, *protC*_*45-90*_ is protein consumption (food consumption on a dry basis* % protein) on a dry mass basis (g) during the recovery period, *lipC* is lipids consumption (food consumption on a dry basis * % lipids) on a dry mass basis (g) during the recovery period.

All values were expressed as mean ± standard error. Differences among means were considered significant at *P*< 0.05. The statistical analyses were performed using INFOSTAT software (Infostat version 2014, Grupo Infostat, FCA-UNC, Argentina).

The survival was tested using the Chi-square test. A two-way ANOVA in a completely randomized design was used to evaluate the effect of the temperature (fixed factor with three levels: 23±1°C, 27±1°C and 31±1°C), and the feeding regime (fixed factor with two levels: control-DF and cyclic feeding-CF) on the following variables: body mass, SGR, AFE, PER, LER, at the end of restriction and recovery periods. A three-way ANOVA in a completely randomized design was used to evaluate the effect of the temperature (fixed factor with three levels: 23±1°C, 27±1°C and 31±1°C), feeding regime (fixed factor with two levels: control-DF and cyclic feeding-CF) and time (fixed factor with five levels: days 1, 45, 60, 75 and 90) on the following variables: HSI, RPM and energetic reserves. Data were tested for normality and homogeneity of variance using the Shapiro-Wilks test and Levene's F-test, respectively, and when necessary, data were log-transformed or arcsine-transformed to meet assumptions. When the assumptions were not met, a method for analysis of ranked data, which is an extension of the non-parametric Kruskal-Wallis test was applied [25]. For multiple comparisons, the LSD test was used.

A factorial ANOVA in a Repeated Measure design using mixed models was used to evaluate the effect of the temperature (fixed factor with three levels: 23±1°C, 27±1°C and 31±1°C), the feeding regime (fixed factor with two levels: control-DF and cyclic feeding-CF), the time (fixed factor with three levels: 45–60, 60–75 and 75–90) and individuals (experimental unit; random factor) during recovery period on FI. When suitable, LSD test was used for multiple comparisons.

## Results

### Survival

At the end of the restriction period (day 45), survival (based on the initial 50 replicates) was similar among treatments (*χ* ^2^_5_ = 7.16; *P* = 0.2093), with an average of 96%. At the end of the recovery period (day 90), survival (based on the remaining juveniles that were not selected for the analysis of organ-somatic indexes at days 45, 60 and 75) was also similar among treatments (*χ* ^2^_5_ = 9.19; *P* = 0.084), with an average of 90%.

### Compensatory and catch-up growth

**Fig 2:**
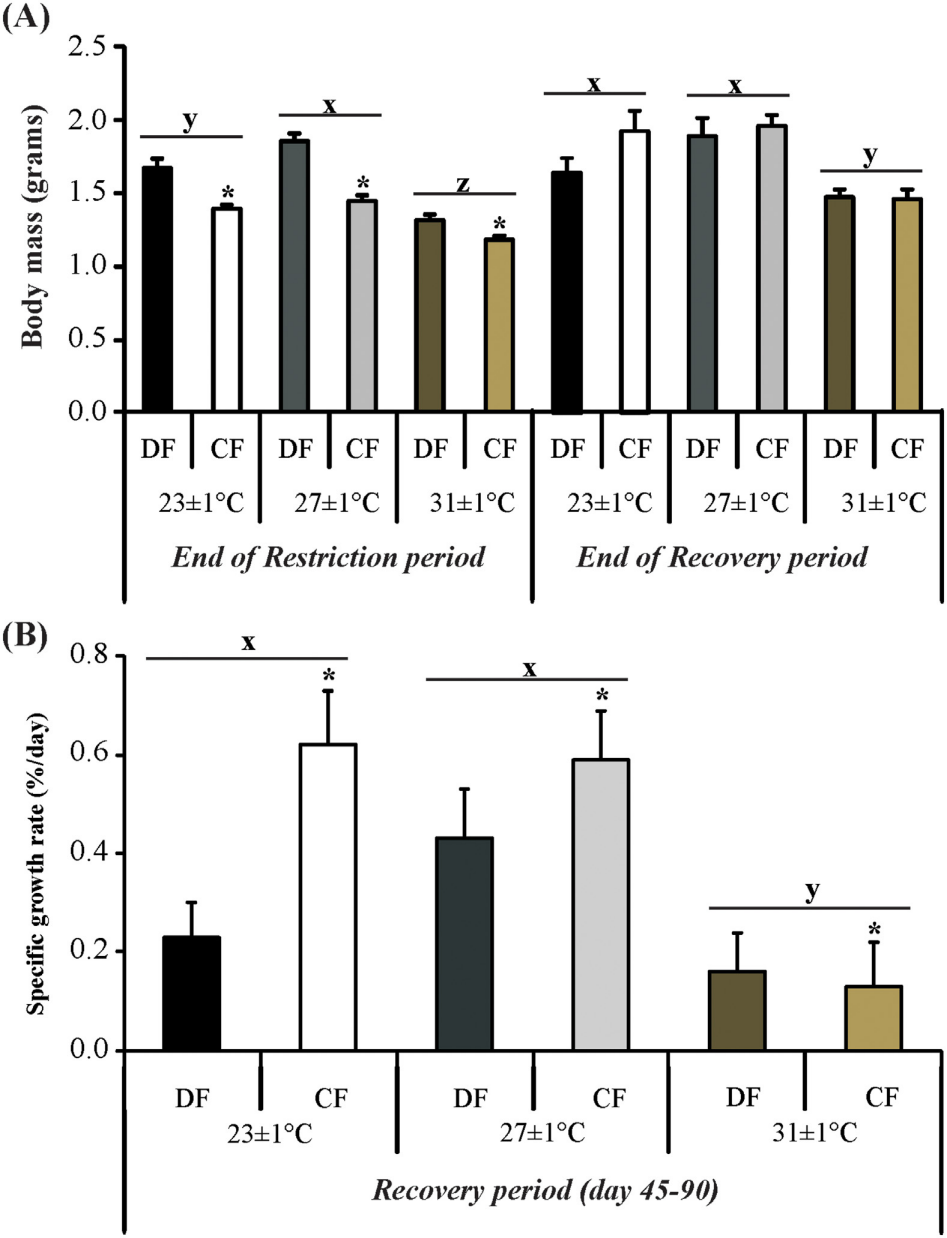
Growth of the redclaw crayfish *C*. *quadricarinatus*. (A) Body mass of the juveniles after the end of the restriction period and after the end of the recovery period. (B) Specific growth rate of the redclaw crayfish *C*. *quadricarinatus* after 45 days of daily feeding (recovery period). Temperature regimes: 23±1°C, 27±1°C and 31±1°C and feeding regimes: DF (juveniles fed daily throughout the experimental period) and CF (juveniles fed for 4 days followed by 4 days of food deprivation, intermittently during the first 45 days of the experimental period, and fed daily from day 45 to day 90). Letters “x,y,z” indicate significant differences among temperatures. Asterisk indicates significant differences between feeding regimes.

Growth performance of crayfish at the end of both periods is presented in Fig 2. At the end of restriction feeding period, body mass of juveniles was significantly affected by feeding regime (*F*_1, 283_ = 56.11; *P*< 0.001), and temperature (*F*_2,283_ = 51.01; *P*< 0.001) (Fig 2A). Body mass of juveniles in CF regime was lower than that of juveniles in DF regime and body mass of crayfish exposed to low and high temperatures was lower than that of crayfish exposed to optimum temperature. After a recovery period of 45 days, the effect of temperature on body mass persisted (*F*_2,74_ = 13.26;*P*< 0.001), in which the weight of juveniles exposed to 31±1°C was lower than that of juveniles exposed to 23±1°C and 27±1°C, whereas the body mass was not statistically different between feeding regimes (*F*_1,74_ = 1.73; *P* = 0.192) (Fig 2A).

Specific growth rate (SGR) during the recovery period was significantly affected by feeding regime (*F*_1, 68_ = 8.66; *P* = 0.004) and temperature (*F*_2, 68_ = 4.00_;_ *P* = 0.023). This variable was higher in juveniles cyclically fed than those daily fed at all temperatures (Fig 2B). The lower growth was in juveniles exposed to the high temperature.

### Hyperphagia and food efficiency

**Fig 3:**
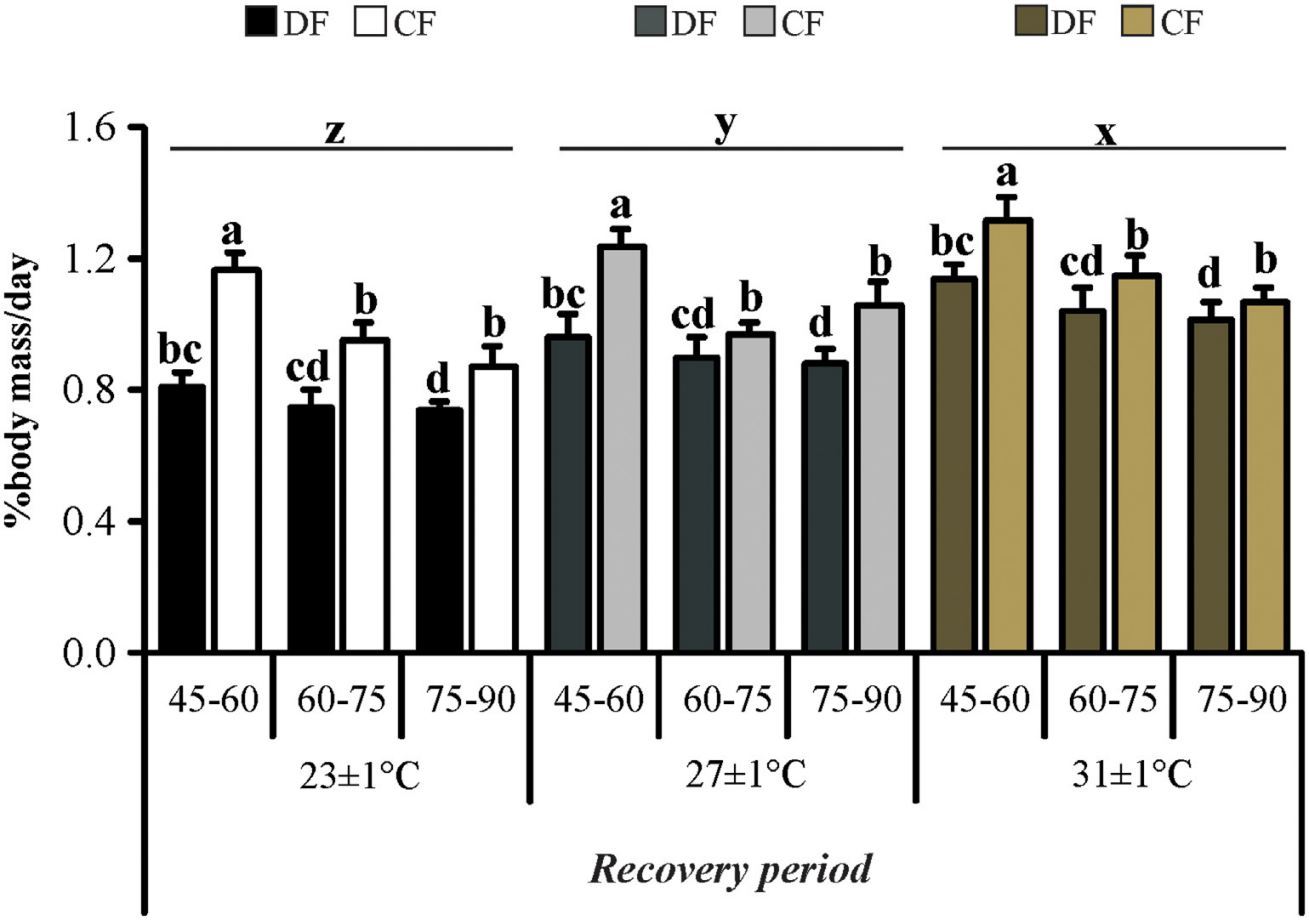
Feeding intake of the redclaw crayfish *C*. *quadricarinatus* during the recovery period. Temperature regimes: 23±1°C, 27±1°C and 31±1°C; feeding regimes: DF (juveniles fed daily throughout the experimental period) and CF (juveniles fed for 4 days followed by 4 days of food deprivation in cycles during the first 45 days of the experimental period, and fed daily from day 45 to day 90), and different time of the recovery period: days 45–60, days 60–75 and days 75–90. Letters “a,b,c,d” indicate significant differences interaction between feeding regime and time. Letters “x,y,z” indicate significant differences among temperatures.

The feeding intake (in terms of % of body mass *per* day) during the recovery period was affected by interaction between feeding regime and time (*F*_2, 148_ = 3.61_;_ *P* = 0.029) and by temperature (*F*_2, 74_ = 20.54; *P*< 0.001) (Fig 3). Hyperphagia was noticed in juveniles under CF regime at all times, and these juveniles consumed ~ 27%, 14% and 17% more food than those non-restricted in the first, second and third fortnight, respectively. On the other hand, juveniles exposed to a higher temperature had superior consumption, in proportion of their body, followed by optimum and low temperatures.

The highest efficiencies in food (*F*_1, 74_ = 4.67; *P* = 0.034), protein (*F*_1, 74_ = 4.70; *P* = 0.033) and lipids (*F*_1, 74_ = 4.73; *P* = 0.033) intakes were noticed in juveniles under CF regime after 45 days of recovery period (Table 1). On the other hand, juveniles exposed to a higher temperature had lower food efficiency (*F*_2, 74_ = 5.82; *P* = 0.004), proteins efficiency (*F*_2, 74_ = 5.86; *P* = 0.004) and lipids efficiency (*F*_2, 74_ = 5.87; *P* = 0.004), than those exposed to optimum and low temperatures.

**Table 1:** Food efficiency of the redclaw crayfish *Cherax quadricarinatus* after recovery period ^1^ ^,^ ^2^ ^,^ ^3^ ^,^ ^4^.

		AFE	PER	LER
**23 ± 1°C**	**DF**	0.38 ± 0.12 **x**	0.80 ± 0.25 **x**	5.81 ± 01.84 **x**
**23 ± 1°C**	**CF**	0.74 ± 0.12 **x** *	1.56 ± 0.24 **x** *	11.42 ± 1.79 **x** *
**27 ± 1°C**	**DF**	0.48 ± 0.10 **x**	1.02 ± 0.21 **x**	7.45 ± 1.56 **x**
**27 ± 1°C**	**CF**	0.60 ± 0.10 **x**	1.26 ± 0.22 **x** *	9.21 ± 1.61 **x** *
**31 ± 1°C**	**DF**	0.22 ± 0.07 **y** *	0.46 ± 0.14 **y**	3.38 ± 1.03 **y**
**31 ± 1°C**	**CF**	0.28 ± 0.09 **y**	0.59 ± 0.18 **y** *	4.29 ± 1.32 **y** *

^1^ AFE = apparent food efficiency; LER = apparent lipids efficiency ratio; PER = apparent protein efficiency ratio.

^2^ Temperature regimes: 23±1°C, 27±1°C and 31±1°C and feeding regimes: DF (juveniles fed daily throughout the experimental period) and CF (juveniles fed for 4 days followed by 4 days of food deprivation, intermittently during the first 45 days of the experimental period, and fed daily from day 45 to day 90).

^3^ Letters “x,y” indicate significant differences among temperatures.

^4^ Asterisk indicates significant differences between feeding regimes.

### Organ-somatic indexes, biochemical and histological analysis

**Fig 4:**
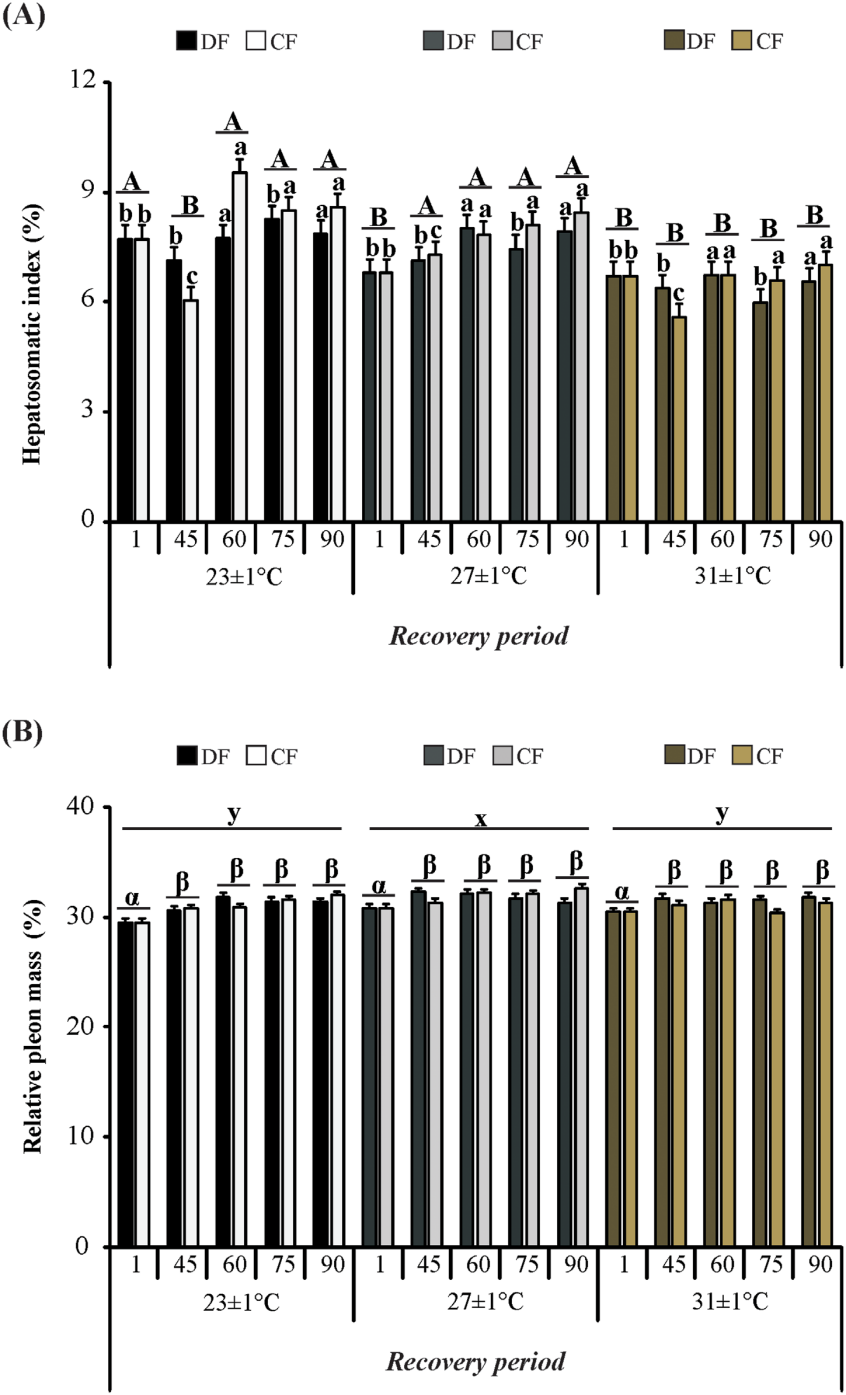
Organ-somatic indexes of the redclaw crayfish *C*. *quadricarinatus* throughout the experiment. (A) Hepatosomatic index and (B) Relative pleon mass. Temperature regimes: 23±1°C, 27±1°C and 31±1°C; feeding regimes: DF (juveniles fed daily throughout the experimental period) and CF (juveniles fed for 4 days followed by 4 days of food deprivation intermittently during the first 45 days of the experimental period and fed daily from day 45 to day 90), and days of the experiment: day 1 (beginning of the experiment), day 45 (the end of restriction period), day 60, 75 and 90 (recovery period). Letters “a,b,c” indicate significant differences interaction between feeding regime and time. Letters “A,B” indicate significant differences interaction between temperature and time. Letters “x,y,z” indicate significant differences among temperatures. Letters “α,β” indicate significant differences among time.

The effects of interaction of feeding regime and time, and the effects of interaction of temperature and time (*F*_8, 239_ = 2.72; *P* = 0.007) on hepatosomatic index (*F*_4, 239_ = 2.74; *P* = 0.029) are shown in Fig 4A. At the end of restriction period, HSI was lower in juveniles of CF regime. However, with the shift to the daily feeding, the HSI was equated to the level of DF regime at day 60, and at day 75 these juveniles had higher HSI than juveniles of DF, accordingly this increased value remained until the end of the experiment (although not statistically significant). On the other hand, after 1 week of acclimatization period, juveniles exposed to 23°C had higher HSI than juveniles exposed to 27°C and 31°C. In turn, at day 45 this value was higher in juveniles exposed to 27°C. At day 60, HSI of juveniles at 23°C was equated to the value of juveniles at 27°C, and these were higher than juveniles at 31°C until the end of the experiment.

The relative pleon mass was affected by temperature (*F*_2, 239_ = 4.91; *P* = 0.008) and time (*F*_4, 239_ = 6.43; *P*< 0.001), and these effects are shown in Fig 4B. The lower value of RPM was observed at the beginning of the experiment (day 1), but at day 45 this value increased and remained stable until the end of the experiment (day 90). On the other hand, higher values were recorded in juveniles exposed to 27°C when compared to juveniles exposed to 23°C and 31°C.

**Fig 5:**
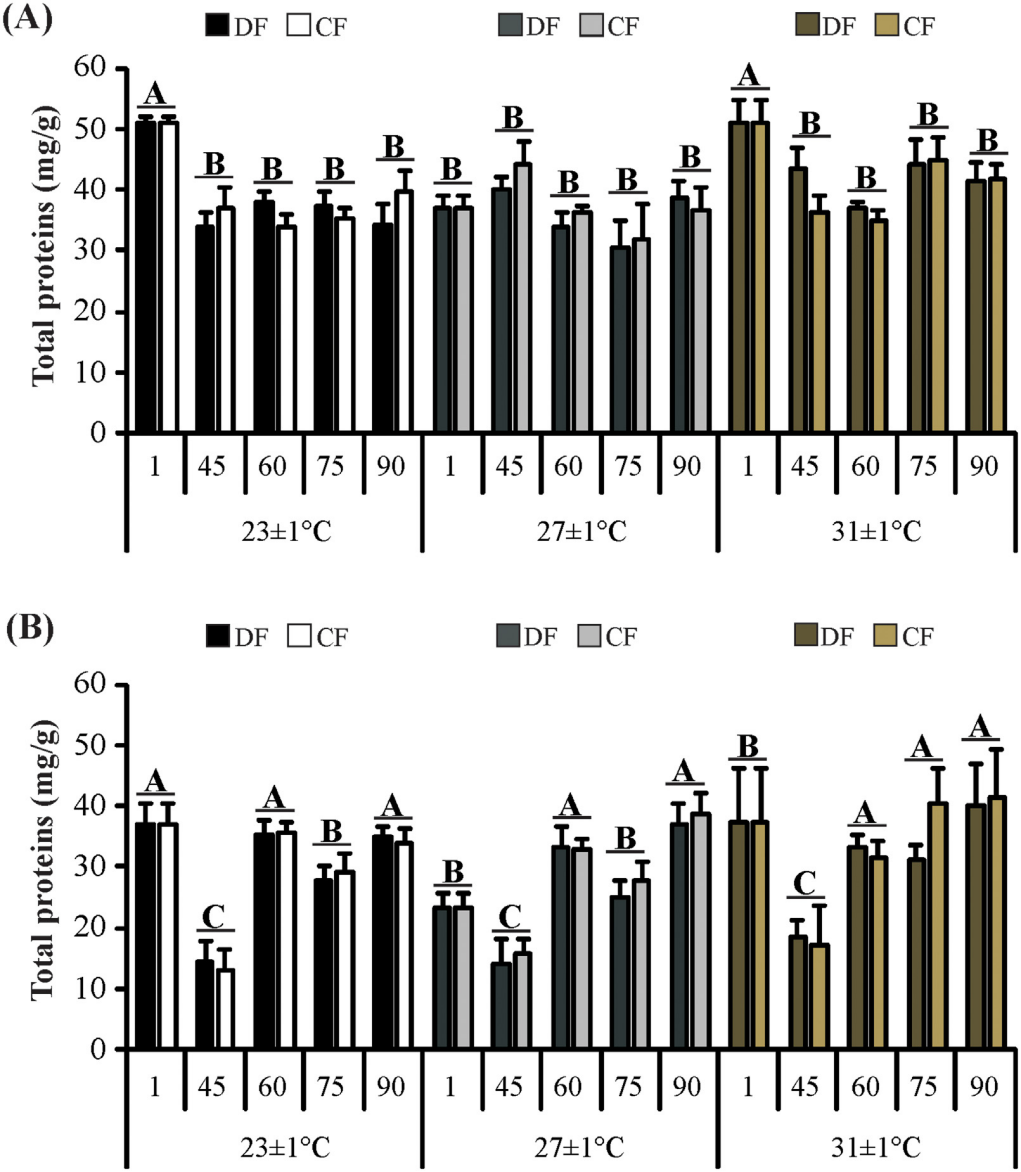
Total proteins in the tissues of the redclaw crayfish *C*. *quadricarinatus* throughout the experiment. (A) Hepatosomatic index and (B) Relative pleon mass. Temperature regimes: 23±1°C, 27±1°C and 31±1°C; feeding regimes: DF (juveniles fed daily throughout the experimental period) and CF (juveniles fed for 4 days followed by 4 days of food deprivation intermittently during the first 45 days of the experimental period and fed daily from day 45 to day 90), and days of the experiment: day 1 (beginning of the experiment), day 45 (the end of restriction period), day 60, 75 and 90 (recovery period). Letters “A,B,C” indicate significant differences interaction between temperature and time.

Regarding the biochemical analysis, an effect of the interaction between temperature and time on concentration of total proteins in hepatopancreas (*F*_8, 97_ = 3.76; *P*< 0.001) and abdominal muscle (*F*_8, 124_ = 2.77; *P* = 0.007) was observed. In the hepatopancreas, juveniles acclimated to 23°C and 31°C had higher total proteins values at the beginning of the experiment (day 1) that those juveniles exposed to 27°C. However, in the remaining of the experiment the values were similar at all temperatures tested (Fig 5A). In the abdominal muscle, juveniles acclimated to 23°C had higher total proteins values at the beginning of the experiment (day 1) that those juveniles exposed to 27°C and 31°C, while at day 45 these values were similar among temperatures. At day 75, juveniles exposed to 31°C had a higher protein concentration than those juveniles exposed to 23°C and 27°C, but at the end of the experiment these values were equated (Fig 5B).

**Fig 6:**
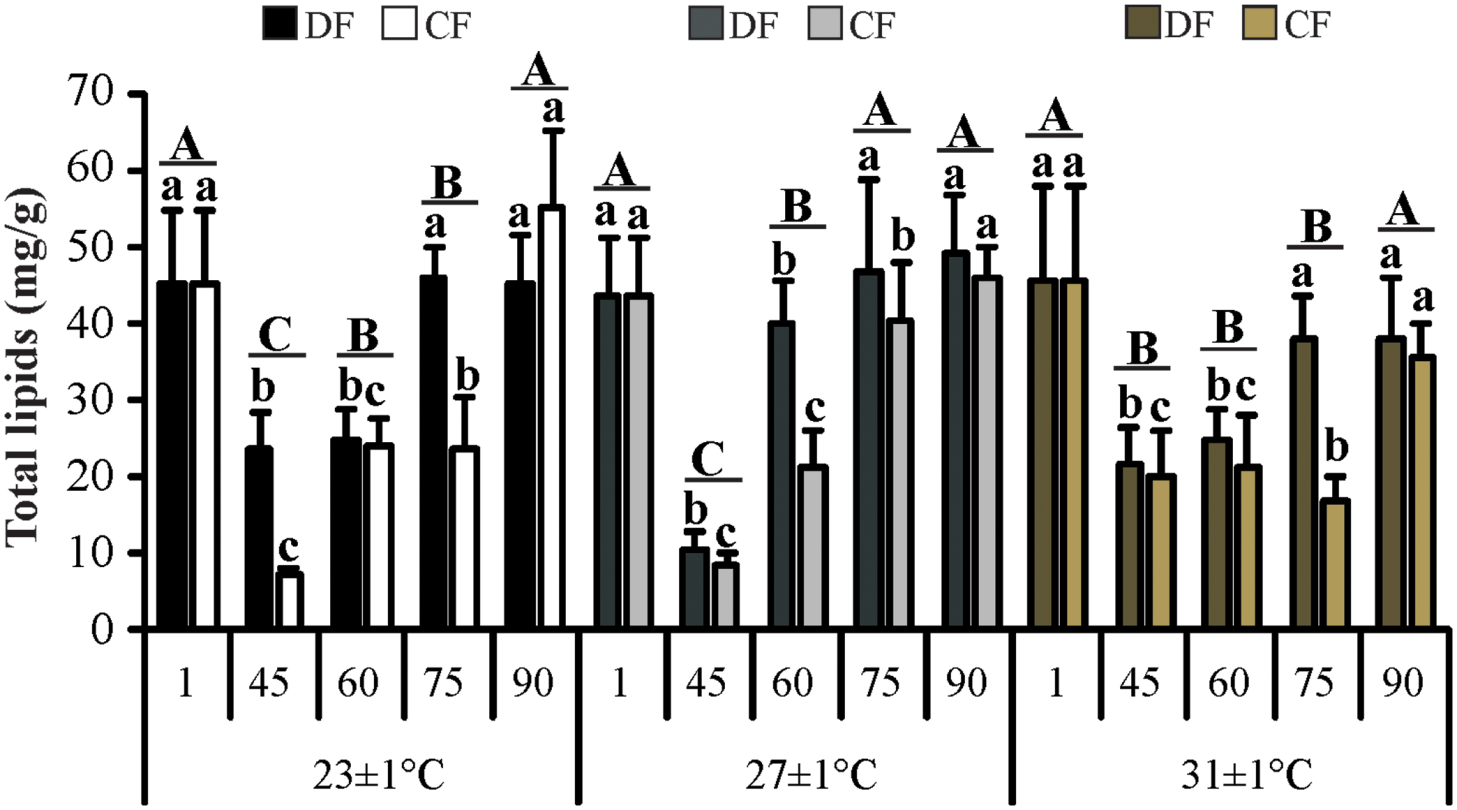
Total lipids in the hepatopancreas of the redclaw crayfish *C*. *quadricarinatus* throughout the experiment. Temperature regimes: 23±1°C, 27±1°C and 31±1°C; feeding regimes: DF (juveniles fed daily throughout the experimental period) and CF (juveniles fed for 4 days followed by 4 days of food deprivation, intermittently during the first 45 days of the experimental period, and fed daily from day 45 to day 90), and days of the experiment: day 1 (beginning of the experiment), day 45 (the end of restriction period), day 60, 75 and 90 (recovery period). Letters “a,b,c” indicate significant differences interaction between feeding regime and time. Letters “A,B,C” indicate significant differences interaction between temperature and time.

Hepatopancreas total lipids concentration, was affected by the interaction between feeding regime and time (*F*_4, 99_ = 2.76; *P* = 0.032), and between temperature and time (*F*_8, 99_ = 2.80; *P* = 0.007) (Fig 6). A lower concentration of this nutrient in juveniles of CF regime was observed at the end of the restriction period (day 45), and during the first thirty days of the recovery period (days 60 and 75). However, the concentration of total lipids was fully recovered in the CF regime at the end of the experiment. At all temperatures tested, the juveniles began the experiment showing a high concentration of total lipids in the hepatopancreas but this value declined at day 45. Finally, the juveniles reached the initial levels of lipids at the end of the experiment.

Regarding total lipids concentration in the abdominal muscle, time showed an effect (*F*_4, 92_ = 27.21; *P*< 0.001), where juveniles exhibited higher concentrations at the beginning of the experiment (day 1), and over time, by the end of the experiment this value was reduced to 70%.

**Fig 7:**
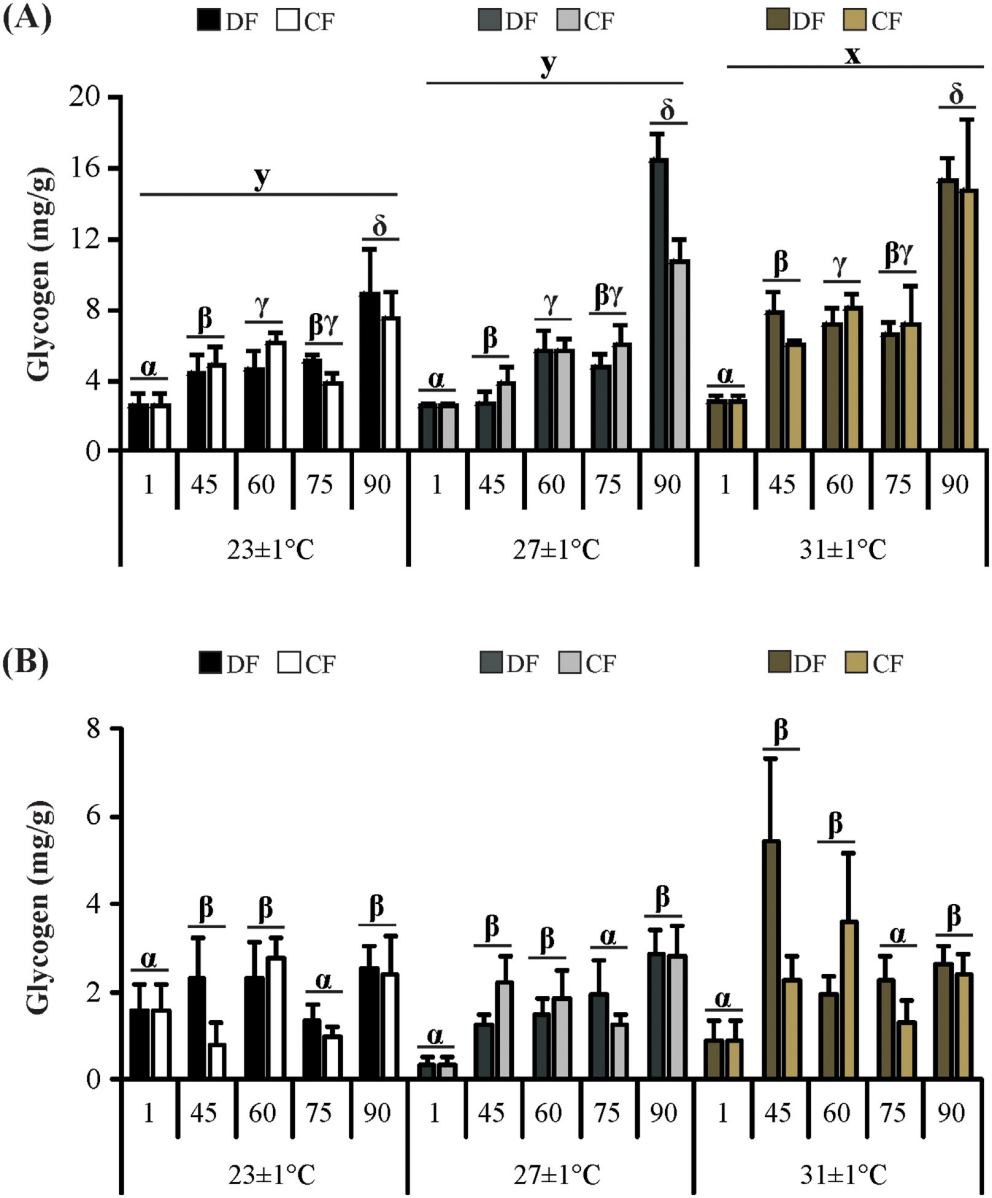
Glycogen in the tissues of the redclaw crayfish *C*. *quadricarinatus* throughout the experiment. (A) Hepatosomatic index and (B) Relative pleon mass. Temperature regimes: 23±1°C, 27±1°C and 31±1°C; feeding regimes: DF (juveniles fed daily throughout the experimental period) and CF (juveniles fed for 4 days followed by 4 days of food deprivation, intermittently during the first 45 days of the experimental period, and fed daily from day 45 to day 90), and days of the experiment: day 1 (beginning of the experiment), day 45 (the end of restriction period), day 60, 75 and 90 (recovery period). Letters “x,y” indicate significant differences among temperatures. Letters “α,β,γ,δ” indicate significant differences among time.

In the hepatopancreas, glycogen concentration was affected by temperature (*H* = 9.20; *P* = 0.010) and time (*H* = 59.53; *P*< 0.001). The highest values were found in juveniles at 31°C, and in juveniles at the end of the experiment (Fig 7A). In the abdominal muscle, concentration was affected by time (*H* = 23.85; *P*< 0.001) (Fig 7B).

**Fig 8:**
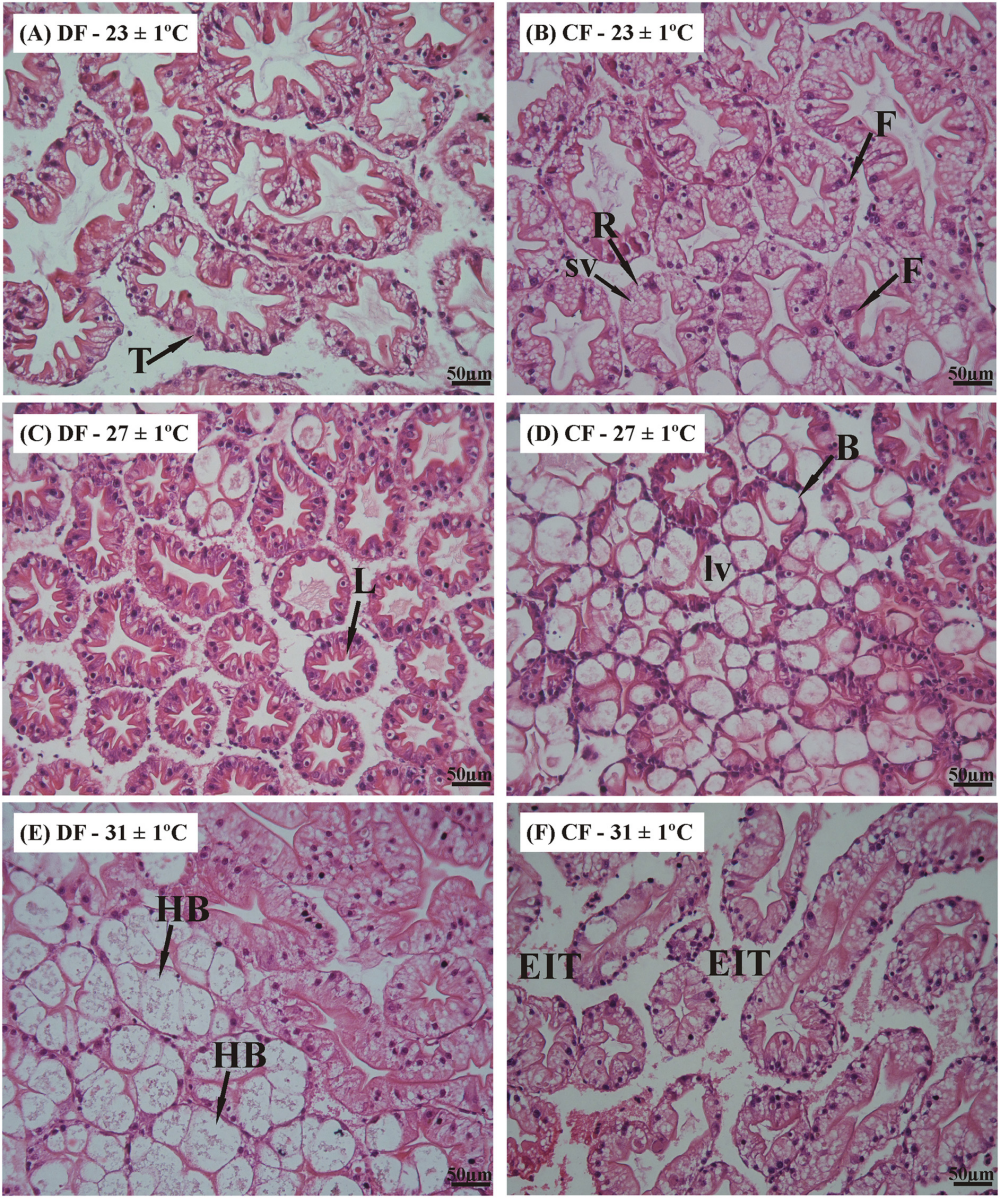
Histological sections of the hepatopancreas in the redclaw crayfish *C*. *quadricarinatus* at the end of restriction period. Temperature regimes: 23±1°C, 27±1°C and 31±1°C; feeding regimes: DF (juveniles fed daily throughout the experimental period) and CF (juveniles fed for 4 days followed by 4 days of food deprivation, intermittently during the first 45 days of the experimental period, and fed daily from day 45 to day 90). B: B-cell; F: F-cell; HB: hypertrophy of B-cell; Is: intertubular space; L: lumen; Lv: large vacuole from B-cell; R: R-cell; Sv: Small vacuole from R-cell; T: tubular structure.

**Fig 9:**
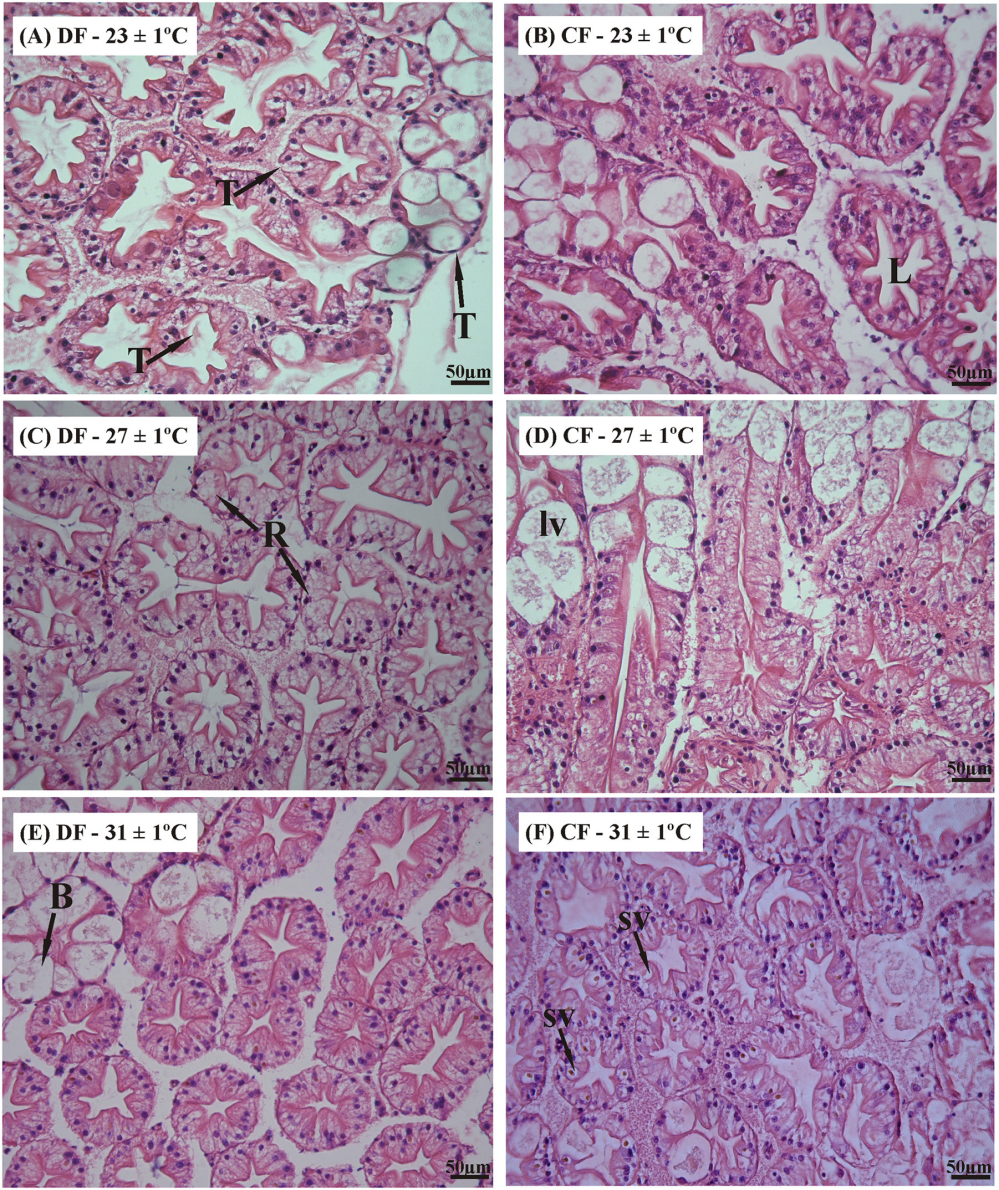
Histological sections of the hepatopancreas in the redclaw crayfish *C*. *quadricarinatus* at the end of recovery period. Temperature regimes: 23±1°C, 27±1°C and 31±1°C; feeding regimes: DF (juveniles fed daily throughout the experimental period) and CF (juveniles fed for 4 days followed by 4 days of food deprivation, v during the first 45 days of the experimental period, and fed daily from day 45 to day 90). B: B-cell; F: L: lumen; Lv: large vacuole from B-cell; R: R-cell; Sv: Small vacuole from R-cell; T: tubular structure.

The hepatopancreas was characterized based on recent descriptions of this digestive gland for this species [24]. The structure of the hepatopancreas of *C*. *quadricarinatus* resembles that of other decapod crustaceans and it is composed of numerous blinded tubules with four main cell types, i.e., E-, F-, B- and R-cells [24,26] (Fig 8). Histological observation of the hepatopancreas revealed no structural differences between the CF and DF regimens at the end of restriction and recovery periods, when the juveniles were exposed at 23°C and 27°C (Figs 8A–8D and 9A–9D). However, at the end of restriction period in juveniles of DF regime at 31°C was observed a hypertrophy of B- cells (Fig 8E), and in juveniles of CF regime at 31°C was observed a greater inter-tubular space (Fig 8F), but these abnormalities disappeared at the end of recovery period (Fig 9E and 9F).

## Discussion

The exploration of compensatory growth with complete catch-up in aquaculture may bring many advantages because it may allow producers to reduce the amount of food provided and consequently reduce feed costs, improve water quality by minimizing food waste, increase food utilization and growth efficiency, and reduce manpower [1,27,28].

This was the first study which determined the effect of temperature on recovery growth of redclaw crayfish and the mechanisms involved in their compensatory response. After 45 days of daily feeding resumption, the previously restricted crayfish held at 23, 27, and 31 ± 1°C displayed a complete body weight catch-up compared to their respective controls, through compensatory growth. The recovery growth was achieved through hyperphagia and improvement in apparent food efficiency. These two mechanisms have been proposed to be the physiological basis of compensatory response, which can occur simultaneously or not [1, 27, 29–33]. In crustaceans there is no record that hyperphagia and improvement of food efficiency have occurred simultaneously during growth recovery, nevertheless studies done in the Chinese shrimp *F*. *chinensis* to stimulate recovery growth, showed that the compensatory response with complete catch-up was achieved through the hyperphagia, but improved food efficiency was not observed [8, 34–36]. In the Pacific white shrimp *Litopenaeus vannamei* the compensatory response with complete catch-up was achieved through the improvement of feed efficiency by Zhu et al. (2014) [37], and in the absence of improved food efficiency by Wasielesky Jr. et al. (2014) [38], but hyperphagia was not measured in both cases. In the Malaysian prawn *Macrobrachium rosenbergii*, the compensatory response with partial and complete catch-up was achieved through the improved food efficiency, but hyperphagia was not measured [39, 40,]. Nevertheless, narrow-clawed crayfish *Astacus leptodactylus* could fully compensate growth, but no food efficiency improvement was observed, and hyperphagia was not measured at this time [41].

In the present study juveniles of *C*. *quadricarinatus* reared under cyclic feeding regime and the three assayed temperatures, exhibited a markedly higher food intake evidencing hyperphagia and this response lasted throughout the recovery phase. Moreover, apparent food efficiency was also noticeable. So, the hyperphagic response could allow a high growth rate, and the apparent food efficiency improvement could be a result of a greater energetic efficiency caused by a lower metabolic expenditure, a persistent feature of the restriction period [8, 42, 43]. Regarding the measurement of metabolic rate, further studies might show the association between food efficiency, food intake and specific growth rate, and metabolism adjustment.

Results of the current study suggest that energy provided to juveniles under cyclic feeding regime during the restriction period was at the expense of hepatopancreatic lipids and recovery of such impact occurred after 45 days of daily feeding. The use of lipids reserves as a metabolic fuel was verified in others species of crustaceans such as prawn *Penaeus esculentus*, lobster *Nephrops norvegicus* exposed to short starvation period [44,45], and in the Chinese mitten-handed crab *Eriocheir sinensis* and redclaw crayfish *C*. *quadricarinatus* exposed to a long starvation period [46,47]. Interestingly, it was the first time that it was observed the utilization of an endogenous reserve, in the redclaw crayfish exposed to moderate restriction, when compared with other studies done by Stumpf et al. (2014a, 2014 b) [5,26]. However, at the end of the experiment similar values between feeding regimes for reserves of hepatopancreatic lipids were observed, indicating that compensatory growth with complete catch-up was also associated with full recovery of this nutrient.

The higher hepatosomatic index in juveniles of CF regime during recovery period could be related with lower concentration of lipids in this phase, suggesting that due to lipids catabolism, metabolic water was released and this water or external water accumulated in this tissue so as to maintain the internal turgidity [48–50].

On the other hand, no small vacuoles were observed in the R cells, which are responsible for lipid storage [51] (at least for the topographical technique used in this study). Therefore, an assessment of the area of digestive cell [52] or histochemical analysis [53] would be the most appropriate analysis to this issue.

Temperatures tested in this study are within the range of temperatures (16°C to 32°C) that allow positive growth and survival for adults and advanced juveniles of *Cherax quadricarinatus* [12]. Tropea et al. (2010) [17] did not observe a high temperature effect (31°C) on female’s growth after a long-term (from an early juvenile ~ 0.200 g to the adult stage), but they observed significant reduction in male growth. In turn, in the present study, temperature had a great effect on growth, feed intake, food efficiency and metabolism of the juvenile crayfish, which grew less when exposed to 31±1°C during both periods and were less efficient with food utilization, although more food was ingested in terms of % of body mass.

Growth at 31±1°C was drastically reduced and this result agreed with a lower efficiency of proteins and lipids ingested. Increased temperature reduces nutrient utilization such as proteins and lipids in brook trout *Salvelinus fontinalis* as observed by Amin et al. (2014) [54]. Crayfishes *Procambarus clarkii* and *P*. *zonangalus* when exposed to 32°C showed an increase in the carbohydrate utilization and possibly this response was a consequence of energy requirement for maintenance, related to changes in metabolic rate which are usually dependent on the ambient temperature [7]. In addition, Powell and Watts (2010) [55] also observed in *P*. *clarkii* and *P*. *zonangalus* a greater ability to absorb this source of cheap energy required for their high metabolism when exposed to high temperatures. Hence, this would be an explanation consistent with our results for the high values of glycogen in the hepatopancreas found throughout the experiment in juveniles exposed to 31±1°C.

The optimal growth of *C*. *quadricarinatus* is reported at 27±1°C, which correlates with optimal growth performance and food efficiency verified in this study. Interestingly at a low temperature such as 23±1°C, the growth at the end of the experiment was similar to the optimum temperature, but with a smaller amount of food consumed and equal food efficiency. These results suggest that this restriction period (CF regime by 45 days) can be used as an important tool to promote growth in conditions of low temperature with no obstacles for further growth.

## Conclusion

In conclusion, *C*. *quadricarinatus* 1-g juveniles maintained individually and at temperatures 23, 27, and 31±1°C showed recovery growth at the end of a 45-day period of daily feeding that followed a 45-day period of cyclic feeding. This physiological response may be explained by an increase in food intake and an improvement in the food efficiency during the recovery period. Temperature appears to have an effect on absorption of macronutrients and subsequent allocation to metabolic activities (growth *versus* maintenance), hence further analysis evaluating nutrient retention would be appropriate to verify the relationship between absorption and utilization of nutrients.

## Supporting Information

S1 Table Number of replicates used in each treatment for analysis of organ-somatic indexes, biochemical analysis of hepatopancreas and abdominal muscle at the start of the experiment (day 1), at the end of restriction period (day 45), and during recovery period (days 60, 75 and 90) ^1^. ^1^ Temperature regimes: 23±1°C, 27±1°C and 31±1°C and feeding regimes: DF (juveniles fed daily throughout the experimental period) and CF (juveniles fed for 4 days followed by 4 days of food deprivation, intermittently during the first 45 days of the experimental period, and fed daily from day 45 to day 90).
(PDF)
