# Supplementary material for: Compensatory Growth in Juveniles of Freshwater Redclaw Crayfish Cherax quadricarinatus Reared at Three Different Temperatures: Hyperphagia and Food Efficiency as Primary Mechanisms
Source: PLoS One. 2015 Sep 30;10(9):e0139372. doi: 10.1371/journal.pone.0139372 (PMC4589333; doi:10.1371/journal.pone.0139372)
Supplement: S1 Table — 1 Temperature regimes: 23±1°C, 27±1°C and 31±1°C and feeding regimes: DF (juveniles fed daily throughout the experimental period) and CF (juveniles fed for 4 days followed by 4 days of food deprivation, intermittently during the first 45 days of the experimental period, and fed daily from day 45 to day 90). (PDF) [file pone.0139372.s001.pdf]

S1 Table. Number of replicates used in each treatment for analysis of organo-somatic indexes, biochemical analysis of hepatopancreas and abdominal muscle at the initial of the experiment (day 1), at the end of restriction period (day 45) and during recovery period (days 60, 75 and 90) [1].

		Days of the experiment	**1**	**45**	**60**	**75**	**90**
Organ-somatic indexes							
**hepatosomatic and relative of pleon mass**	**23 ± 1°C**	**DF**	8	9	9	9	9
		**CF**	8	9	9	9	9
	**27 ± 1°C**	**DF**	8	9	9	8	9
		**CF**	8	9	9	9	9
	**31 ± 1°C**	**DF**	9	9	9	10	10
		**CF**	9	9	9	10	10
Biochemical analysis of hepatopancreas							
**Total proteins**	23 ± 1°C	**DF**	3	4	5	5	4
		**CF**	3	4	5	5	5
	27 ± 1°C	**DF**	3	4	5	5	5
		**CF**	3	4	5	5	4
	31 ± 1°C	**DF**	3	4	4	5	5
		**CF**	3	4	4	5	4
**Total lipids**	23 ± 1°C	**DF**	3	4	5	5	5
		**CF**	3	4	5	5	5
	27 ± 1°C	**DF**	3	4	5	5	5
		**CF**	3	4	5	5	5
	31 ± 1°C	**DF**	3	4	5	5	5
		**CF**	3	4	4	5	4
**Glycogen**	23 ± 1°C	**DF**	3	4	4	5	4
		**CF**	3	4	4	5	5
	27 ± 1°C	**DF**	3	4	4	5	4
		**CF**	3	4	4	5	4
	31 ± 1°C	**DF**	3	4	4	4	4
		**CF**	3	4	3	4	3
Biochemical analysis of abdominal muscle							
**Total proteins**	23 ± 1°C	**DF**	6	5	5	5	5
		**CF**	6	5	5	5	5
	27 ± 1°C	**DF**	6	5	5	5	5
		**CF**	6	5	5	5	5

	31 ± 1°C	**DF**	6	5	5	5	5
		**CF**	6	5	5	5	5
**Total lipids**	23 ± 1°C	**DF**	3	5	5	3	5
		**CF**	3	5	5	3	5
	27 ± 1°C	**DF**	3	5	5	4	5
		**CF**	3	5	4	5	5
	31 ± 1°C	**DF**	3	4	5	4	3
		**CF**	3	5	5	3	4
**Glycogen**	23 ± 1°C	**DF**	3	5	5	5	5
		**CF**	3	5	5	4	5
	27 ± 1°C	**DF**	3	5	5	5	5
		**CF**	3	5	5	5	5
	31 ± 1°C	**DF**	3	5	5	5	4
		**CF**	3	5	4	3	4

[1] Temperature regimes: 23±1°C, 27±1°C and 31±1 °C and feeding regimes: DF (juveniles fed daily throughout the experimental period) and CF (juveniles fed for 4 days followed by 4 days of food deprivation in cycles during the first 45 days of the experimental period and fed daily from day 45 to day 90).
